# Comparison of Radiation Response between 2D and 3D Cell Culture Models of Different Human Cancer Cell Lines

**DOI:** 10.3390/cells12030360

**Published:** 2023-01-18

**Authors:** Julia Raitanen, Bernadette Barta, Marcus Hacker, Dietmar Georg, Theresa Balber, Markus Mitterhauser

**Affiliations:** 1 Ludwig Boltzmann Institute Applied Diagnostics, 1090 Vienna, Austria; 2 Department of Biomedical Imaging and Image-Guided Therapy, Division of Nuclear Medicine, Medical University of Vienna, 1090 Vienna, Austria; 3 Vienna Doctoral School of Chemistry (DoSChem), University of Vienna, 1090 Vienna, Austria; 4 Institute of Inorganic Chemistry, Faculty of Chemistry, University of Vienna, 1090 Vienna, Austria; 5 Department of Radiation Oncology, Division of Medical Radiation Physics, Medical University of Vienna, 1090 Vienna, Austria

**Keywords:** radiobiology, radiation oncology, radiosensitivity, external photon beam (X-ray), therapy, human cancer cell lines (PC-3; LNCaP; T-47D), multicellular tumor spheroids (3D), clonogenic assay, resazurin assay, γH2AX

## Abstract

Radiation therapy is one of the most effective tools in cancer therapy. However, success varies individually, necessitating improved understanding of radiobiology. Three-dimensional (3D) tumor spheroids are increasingly gaining attention, being a superior in vitro cancer model compared to 2D cell cultures. This in vitro study aimed at comparing radiation responses in 2D and 3D cell culture models of different human cancer cell lines (PC-3, LNCaP and T-47D) irradiated with varying doses (1, 2, 4, 6, 8 or 20 Gy) of X-ray beams. Radiation response was analyzed by growth analysis, various cell viability assays (e.g., clonogenic assay, resazurin assay) and amount of DNA damage (γH2AX Western Blot). Results showed decreasing cell proliferation with the increase of radiation doses for all cell lines in monolayers and spheroids of LNCaP and T-47D. However, significantly lower radiosensitivity was detected in spheroids, most pronounced in PC-3, evincing radiation resistance of PC-3 spheroids up to 8 Gy and significant growth inhibition only by a dose escalation of 20 Gy. Cell line comparison showed highest radiosensitivity in LNCaP, followed by T-47D and PC-3 in 2D, whereas, in 3D, T-47D showed highest sensitivity. The results substantiate the significant differences in radiobiological response to X-rays between 2D and 3D cell culture models.

## 1. Introduction

Radiation biology often provides the rationale for the implementation of new treatment strategies in clinical practice; hence, it is important to foster the basic understanding of radiation effects. Up until now, mostly (two-dimensional) 2D cell monolayers were used to assess therapeutic efficacy of radiation treatments in basic research. Although this technique has many advantages, being simple, cheap, and highly reproducible, it also comprises limitations when trying to compare obtained results to in vivo tumors. These limitations include unnatural cell shapes, the lack of cell differentiation and cell-to-cell communication, as well as the unnaturally equal exposure of cells to nutrients or drugs [1]. To overcome these limitations, and to revolutionize disease modeling and treatment testing, three-dimensional multicellular tumor spheroids were introduced in the early 1940s by Holtfreter [2,3]. In contrast to monolayer culture, multicellular tumor spheroids mimic in vivo tumors more accurately, due to similar growth behaviors as well as cellular heterogeneity within the spheroid and formation of molecular gradients, among others. In this context, promising results utilizing higher dimensionality models for disease simulations and tumor-targeted therapies have been obtained [4]. Their specific characteristics mainly result from their layered structure, consisting of an inner necrotic core, which is surrounded by a quiescent zone and an outer layer of proliferating cells [3,5,6]. Over the years, different techniques for generating spheroids were investigated [7]. The most common methods are: liquid overlay [8], hanging drop [9], spinner flask [10] and magnetic levitation [11]. Due to simplicity, low costs and homogeneity in spheroid size and shape, the liquid overlay technique was used in this study [12].

In the early 1970s, Durand and Sutherland introduced the so-called contact effect when firstly noting the increased radioresistance of spheroids compared to monolayers after irradiation using V79 Chinese hamster lung cells [13]. Since then, this effect has been demonstrated for a number of other cell lines [14,15,16,17,18,19], with the exception of E.E. melanoma spheroids [20]. In this study, the widely used prostate cancer cell lines PC-3 and LNCaP, as well as the not-yet well described breast cancer cell line T-47D, were assessed. Cells were cultivated in 2D and 3D, and irradiated with varying doses (1, 2, 4, 6, 8 and 20 Gy) of X-ray irradiation. Since the existing radiobiological studies use only a limited number of assays, and generally lack data on long term effects, we utilized a variety of standardized assays to analyze the radiation response not only through the short term, but also through the long term (up to 29 days) effects. The aim of this study was to assess the differences in radiation response between monolayers and spheroids, and thereby determine the radiosensitivity of the different human cancer cell lines. To this, growth analysis, including necrotic core monitoring, and the commonly used clonogenic assay were performed. Cell viability was assessed using trypan blue exclusion assay, Cell Titer Glo^®^ 3D and resazurin assay to complement the data. To further investigate molecular mechanisms, γH2AX was investigated by Western Blot analysis.

## 2. Materials and Methods

### 2.1. Cell Cultures

Cell lines were obtained from the American Type Culture Collection (ATCC). PC-3 and LNCaP cells were cultivated in Roswell Park Memorial Institute (RPMI) 1640 medium supplemented with 10% fetal bovine serum, 2 mM Glutamine and 1% penicillin (10,000 U/mL)/streptomycin (10,000 µg/mL). T-47D cells were cultivated in RPMI 1640 medium supplemented with 10% fetal bovine serum and 4 mM Glutamine. All reagents were obtained from Gibco Life Technologies Ltd. (Paisley, UK). Cells were cultured at 37 °C with 5% CO_2_ and tested routinely for mycoplasma contamination using the MycoAlert™ Mycoplasma Detection Kit (Lonza, Basel, Switzerland), according to the manufacturer’s instructions [21].

### 2.2. Generation of Spheroids

Spheroids were generated using the liquid overlay technique. 2000 cells/well (in 100 µL medium) were seeded into U-bottom 96-well plates, precoated with a thin layer of 1.5% low gelling agarose (Sigma Aldrich, St. Louis, MO, USA). LNCaP and T-47D were cultured scaffold-free, whereas 3% Geltrex^TM^ (LDEV-Free Reduced Growth Factor Basement Membrane Matrix, Gibco, Paisley, UK) was added to the PC-3 cell suspension, followed by centrifugation at 161× *g* for 10 min at 4 °C. During spheroid establishment, any influence of Geltrex^TM^ on radiosensitivity was excluded (see Supplementary Figure S1).

### 2.3. Irradiation Procedure

X-ray irradiation (1, 2, 4, 6, 8 or 20 Gy absorbed dose) was performed at 200 kV, 20 mA with a focus size of 5.5 mm using an YXLON reference irradiator (Maxishot, YXLON International GmbH, Hamburg, Germany) with a 0.5 mm copper filter and a 3 mm aluminum filter. Before exposure, dose rate was measured with a cross calibrated ionization chamber (Semiflex TM31012; PTW, Freiburg, Germany). The average dose rate at the level of the respective tumor model systems was about 1.2 Gy/min [22]. 

### 2.4. Microscopic Growth Analysis

Spheroid growth was observed by both visual inspection and size measurements using an Olympus IMT-2 Inverted Microscope with an XC50 Color Camera (Olympus, Tokyo, Japan) and the cellSens Entry (Version 2.3, Olympus, Tokyo, Japan) software on days 3, 5, 8, 9, 11, 13, 15, 19, 23 and 29 after seeding. Media were renewed once per week. Per experiment, one representative spheroid per cell line and radiation dose was chosen, and a mean value of three diameter measurements was calculated and plotted with Prism Version 8 (GraphPad, San Diego, CA, USA).

### 2.5. Live/Dead Staining—Necrotic Core Examination

Spheroids were washed with 100 µL phosphate buffered saline (PBS), and afterwards incubated with propidium iodide (Sigma-Aldrich, St. Louis, USA; 1:500 in medium) for 5 h 45 min followed by the addition of calcein acetoxymethyl ester (Sigma-Aldrich; 4 mM in dimethyl sulfoxide, 1:800 in medium) with further incubation for 15 min. Spheroids were then transferred to flat-bottom 96-well plates, washed with PBS and imaged under an IMT-2 Inverted fluorescence Microscope (Olympus) equipped with a 100 W Mercury Power Supply (Model BH2-RFL-T3, Olympus) for fluorescence detection. Diameters of total spheroid and necrotic core were measured using the Digimizer software version 5.7.2 (MedCalc Software Ltd, Ostend, Belgium).

### 2.6. Clonogenic Assay

Cell survival and reproductive ability after irradiation were determined by performing standard clonogenic assays [23]. 250 PC-3/T-47D or 1000 LNCaP cells were seeded in 6-well plates prefilled with 2 mL of respective medium. On the following day, plates were irradiated with 1, 2, 4, 6 or 8 Gy. After irradiation, PC-3 cells were further cultured for 7 days, T-47D for 14 days and LNCaP for 21 days, with their media being changed once per week. After the respective culturing periods, cells were washed with PBS, fixed with methanol for 30 min at 4 °C and, after another washing step, stained with crystal violet (Sigma-Aldrich; 1% in water) for 3 min. To assess the reproductive ability, stained colonies were counted using a Fusion FX7 (Vilber Lourmat, Eberhardzell, Germany). Three independent experiments were performed in triplicate for each cell line. Plating efficiency was calculated by dividing the average number of colonies in the control by the number of seeded cells. Plating efficiency was then multiplied with the number of colonies after treatment, resulting in the Surviving fraction (SF). Coefficients (α, β) were determined by linear quadratic curve fitting using Equation (1). D corresponds to radiation dose in Gray (Gy).

SF (D) = exp (−αD − βD^2^) (1)

### 2.7. Trypan Blue Exclusion Assay

Cells were seeded in 6-well plates: 500,000 for evaluation after 0 and 24 h, 100,000 PC-3 or 250,000 LNCaP/T-47D for evaluation after 72 h. After overnight incubation, cells were irradiated with single doses of 2, 4, 8 and 20 Gy. After the aforementioned time points, supernatants, wash fraction and harvested cells were combined and centrifuged for 4 min at 161× *g* at room temperature. The supernatant was discarded, and the cell pellet was resuspended in medium. Single cell suspensions were mixed with Trypan blue (1:1; Gibco) to determine cell viability and cell counts, using the LUNA^TM^ automated cell counter (Logos Biosystems, Anyang, Republic of Korea).

### 2.8. Resazurin Assay

Cells were seeded as monolayers in 6-well plates with respect to their growth rate (2000 PC-3, 60,000 LNCaP and 10,000 T-47D), incubated for 2 days and irradiated with 0, 2, 4 or 8 Gy. Cell monolayers were further cultivated under standardized conditions (see Section 2.1) for up to 19 days. On days 5, 13 and 19, the supernatant and detached cells were combined and the liquid volume to seed a total of 5000 cells was calculated from the control. This volume was seeded for all treatment conditions (in total 200 µL/well) in 96-well plates. The next day, cells were incubated with 20 µL resazurin (110 μg/mL in PBS; TCI, Tokio, Japan) for 17 h. Fluorescence detection was performed using a Synergy HTX multimode reader (Ex590/Em645; Biotek, Winooski, VT, USA). 

In 3D, irradiated spheroids were transferred into flat-bottom 96-well plates and incubated with resazurin (17 h) one day prior to fluorescence detection on days 7, 15 and 21, as described before. 

### 2.9. Cell Titer Glo^®^ 3D

The assay was performed according to the manufacturer’s protocol [24]. On days 6, 14 and 20, control and irradiated spheroids were transferred to white-walled 96-well plates (100 µL medium/well). Cell Titer Glo^®^ 3D reagent (Promega, Madison, WI, USA) was thawed in the fridge overnight before use. The next day, the plate and reagent were equilibrated to room temperature before the addition of 100 µL reagent per well. Afterwards, the plate was vigorously shaken for 5 min using the Synergy HTX multimode reader (Biotek, Winooski, VT, USA) and incubated at room temperature for another 25 min before measurement of luminescence.

### 2.10. Western Blot

The amount of DNA double-strand breaks in 2D and 3D cultures was assessed by determining γH2AX as surrogate. Whole cell lysates of monolayers and spheroids were prepared directly after irradiation. For monolayers, 1 million cells were seeded in petri dishes one day prior to irradiation. Lysates were obtained by mechanical scraping using 300 µL radioimmunoprecipitation assay (RIPA) lysis buffer (Gibco) together with a protease inhibitor (P2714 Sigma Aldrich, 1:10 after reconstitution in 10 mL water), shaking for 30 min on ice before centrifugation at 13,523× *g*, 4 °C for 20 min. For 3D lysates, 20 spheroids were pooled and centrifuged at 135× *g* for 4 min at room temperature. After washing with phosphate buffered saline, 60 µL RIPA lysis buffer and a protease inhibitor were added and further treated as described for monolayers.

Protein concentrations were determined using the Pierce™ BCA Protein Assay Kit (Thermo Fisher Scientific, Waltham, MA, USA). Proteins were separated on Mini-PROTEAN TGX precast Gels (Bio-Rad Laboratories Inc., Berkeley, CA, USA) with a running buffer consisting of 3 g Tris(hydroxymethyl)aminomethane (TRIS), 14,5 g Glycine and 1 g Sodium dodecyl sulfate (SDS), dissolved in distilled water. Semi-dry blotting was performed on a Trans-Blot^®^ TurboTM Transfer System (Bio-Rad) using a Trans-Blot Turbo 5× Transfer Buffer (Bio-Rad) and a nitrocellulose membrane (Amersham^TM^ Protran^TM^ Premium 0.2 µm; GE Healthcare, Chicago, IL, USA). 

Membranes were blocked with 5% bovine serum albumin (BSA; Sigma-Aldrich) in TRIS buffered saline with Tween (TBS-T; Sigma-Aldrich) on a shaker for 1 h and at room temperature. Primary antibody incubation (anti-phospho-Histone H2A.X (Ser139), clone JBW301 (Merck, 1:1000 in 5% BSA in TBS-T), β-Tubulin antibody (Cell Signaling Technology; 1:1000 in 5% BSA in TBS-T) was performed overnight at 4 °C. Incubation with the respective horseradish peroxidase (HRP) conjugated secondary antibodies was performed for 1 h and at room temperature before detection of chemiluminescence (Pierce^TM^ ECL Western Blotting Substrate Kit; Thermo Fisher Scientific, Waltham, MA, USA) and a ChemiDoc Imaging System (Bio-Rad), according to the manufacturer.

Band intensities were determined using ImageJ, ratios were calculated and plotted using the Prism software version 8 (GraphPad).

### 2.11. Statistical Analysis

Data was analyzed using Prism (GraphPad). Data comparison was performed by multiple unpaired *t*-tests. *p*-values < 0.05 were considered statistically significant.

## 3. Results

### 3.1. Cell Viability: Trypan Blue Exclusion Assay

**Figure 1:**
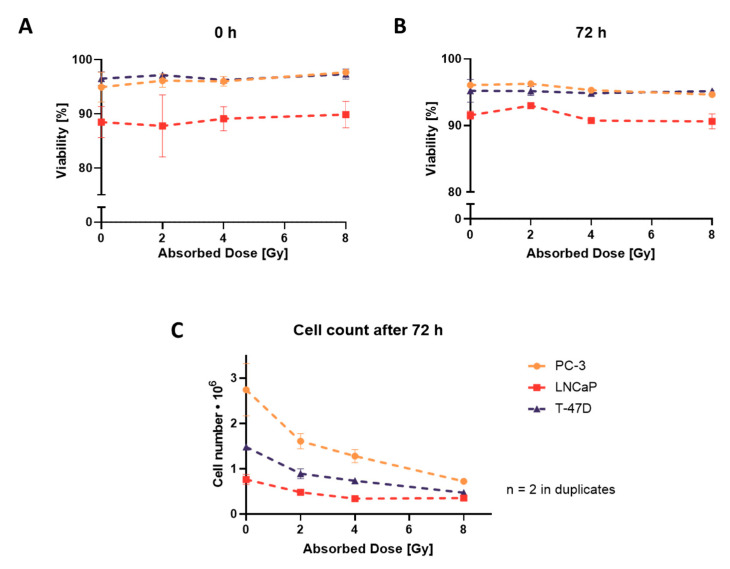
Trypan Blue Exclusion Assay: evaluation of cell viability and proliferation in monolayers. Cell viability immediately (0 h; (**A**)) and 72 h (**B**) after irradiation. (**C**) Cell count 72 h after irradiation. (**A**–**C**) Graphs are plotted as mean ± standard deviation from two independent experiments (*n* = 2) performed in duplicates.

Cell viability in monolayers, evaluated by Trypan Blue Exclusion Assay, showed no significant change in viability when measured immediately (0 h) and 72 h after irradiation up to 8 Gy (Figure 1A,B). However, a decrease in cell numbers with increasing radiation doses was observed 72 h after irradiation (Figure 1C).

### 3.2. Evaluation of Reproductive Potential by Clonogenic Assay

**Figure 2:**
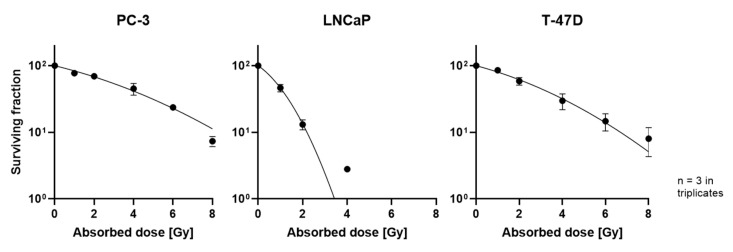
Survival curves of PC-3 (**left**), LNCaP (**middle**) and T-47D (**right**) cells; plotted as mean ± standard deviation from three independent experiments (*n* = 3) performed in triplicate.

To further investigate this effect in monolayers, clonogenic assays for evaluation of reproductive potential were performed. All cell lines showed a decreased number of colonies with increasing radiation dose, albeit to a different extent (Figure 2). Values for 6 and 8 Gy for LNCaP were excluded, since there were no colonies visible.

For better comparability, the coefficients alpha and beta of the survival curve were determined. Higher values correspond to higher radiosensitivity (Table 1), showing highest radiosensitivity in LNCaP, followed by T-47D and PC-3. Representative photographs of assay plates are depicted in the (Supplementary Figure S2).

### 3.3. Growth Analysis

#### 3.3.1. Monolayers

PC-3 monolayers grew with a doubling time of 24 h, whereas LNCaP took 60 h and T-47D 32 h. After irradiation of the LNCaP and T-47D monolayers, further culturing was possible solely for the cells irradiated with 2 or 4 Gy. Irradiation with 8 Gy completely hampered cell proliferation in the two cell lines, as already shown with the clonogenic assay, making further culturing impossible. However, regarding PC-3, even the monolayers irradiated with 8 Gy recovered and regrew.

#### 3.3.2. Spheroids

**Figure 3:**
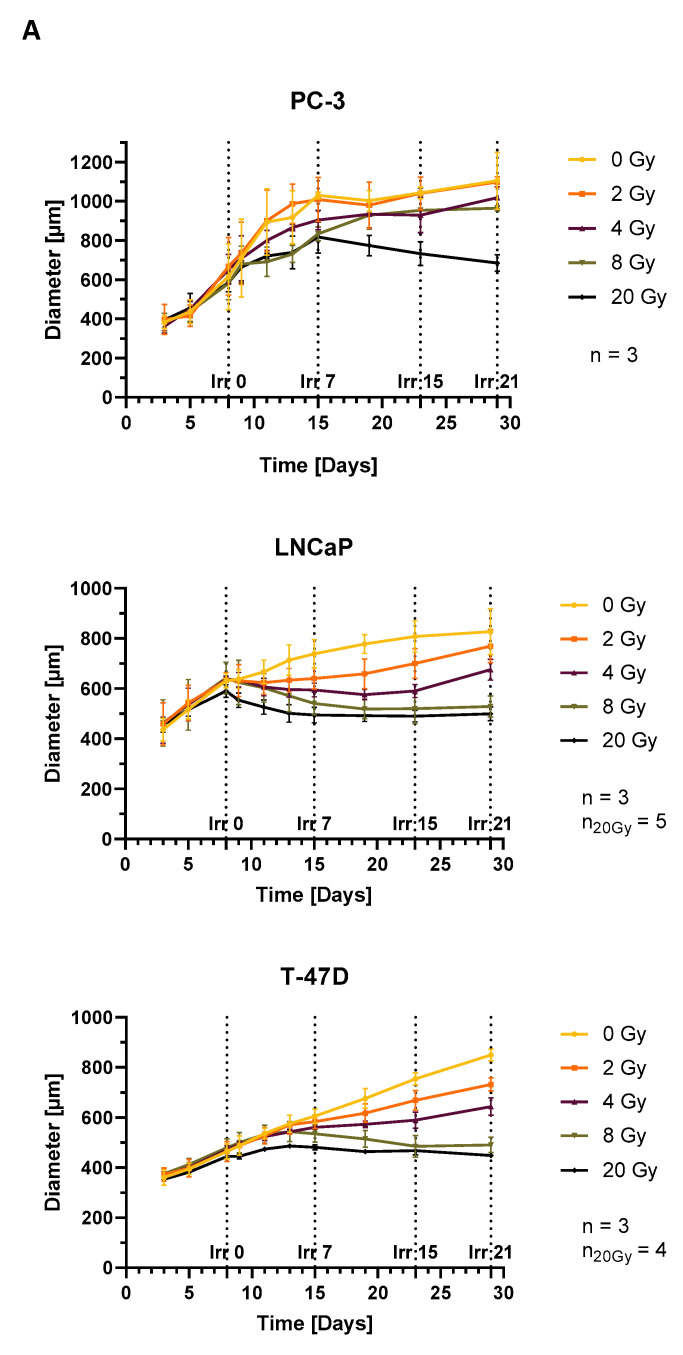

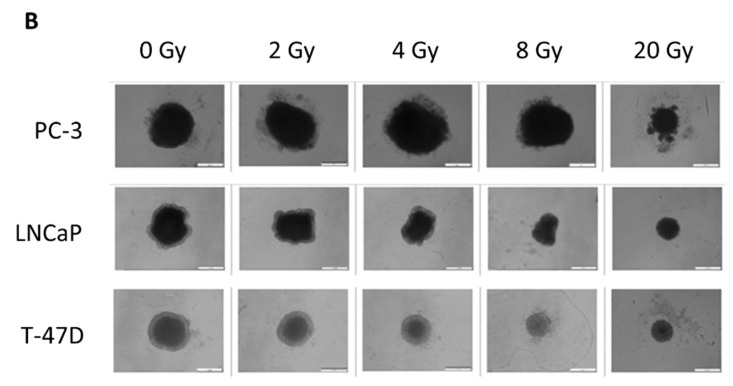
Growth observation of spheroids after single irradiation with 0, 2, 4, 8 or 20 Gy on day 8 (Irr 0); (**A**) Diameter of spheroids; average over three diameter measurements per spheroid and time point plotted as mean ± standard deviation from three to five independent experiments (*n* = 3–5); Irr 0, 7, 15 and 21 indicate the time after irradiation in days (**B**) Growth analysis of spheroids on day 29 (Irr 21); white scale indicates 500 µm.

Spheroid growth was cell line dependent and is shown in Figure 3A. Non irradiated PC-3 spheroids showed exponential growth until day 15, whereas LNCaP and T-47D controls grew rather linearly, LNCaP until day 15 and T-47D until the end of the measured time period. LNCaP and PC-3 reached a plateau at a diameter of around 0.8 and 1 mm, respectively.

Irradiating PC-3 spheroids with 2 Gy showed no significant growth difference to control. However, 4 or 8 Gy radiation initially resulted in a decrease in growth rate, followed by a period of faster growth rate, ultimately leading to no significant size difference to control after 29 days of growth. Only the highest single fraction doses of 20 Gy resulted in a sustained growth inhibition. In contrast, LNCaP and T-47D showed significant size differences to control on day 29 after irradiation with 4 Gy or 2 Gy, respectively. Dose dependent growth inhibition was also shown for higher doses, without regrowth in the observed period after 8 Gy irradiation. Dose escalation to 20 Gy did not result in a significantly stronger growth inhibition compared to 8 Gy, neither for LNCaP nor T-47D spheroids, suggesting 8 Gy already being a highly effective treatment. Disintegration of the spheroids was shown for PC-3 and T-47D after irradiation with 20 Gy. In contrast, LNCaP retained their dense structure and only deformed slightly. Photographs of representative spheroids on day 29 are shown in Figure 3B. Time-lapse images of all types of spheroids described are given in the (Supplementary Figure S3).

### 3.4. Live/Dead Staining

To test whether irradiation directly induces necrosis, live fluorescent imaging was performed. Therefore, spheroids were stained for viable (Acetoxymethyl ester positive; green) and necrotic (PI positive; red) cells.

All spheroid models consisted of a viable outer layer and central necrosis on day 8, except T-47D whose necrotic core was first observed on day 15 of growth. This is in line with previous studies, stating that the spheroid diameter needs to be bigger than 500 µm diameter to develop a necrotic core [8]. However, sizes of necrotic core varied between the cell lines. The ratio of necrotic core to total spheroid size was determined and indicated an increase in the necrotic core ratio with spheroid size, but not with dose, in terms of LNCaP and T-47D up to 8 Gy. Only irradiation with the highest single dose (20 Gy) resulted in higher necrotic core ratios compared to the control in T-47D and PC-3 suggesting induction of necrosis. PC-3 spheroids show neither an increase in the necrotic core ratio with increasing radiation dose, nor with spheroid size up to 8 Gy. These results suggest that there is no additional necrosis induced by irradiation up to 8 Gy. Live staining and semiquantitative assessment are demonstrated in the (Supplementary Figure S4).

### 3.5. Cell Viability Determination Using Cell Titer Glo^®^ 3D

**Figure 4:**
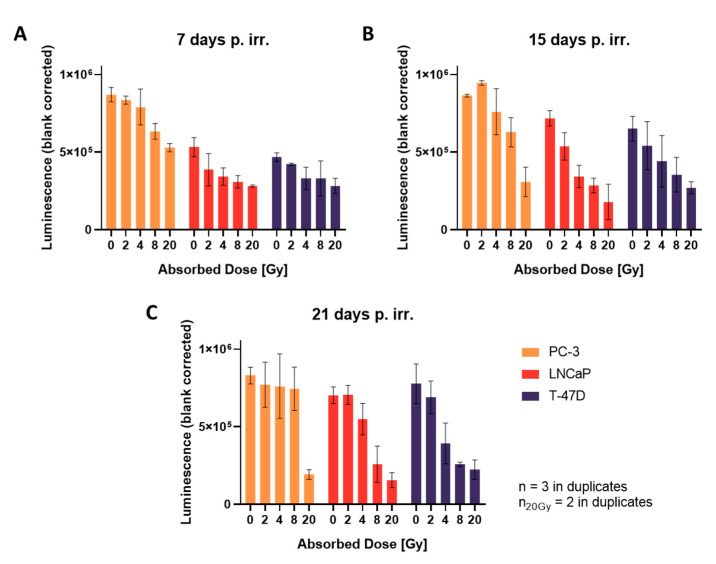
Cell viability of spheroids, (**A**) 7, (**B**) 15 and (**C**) 21 days after irradiation; plotted as mean ± standard deviation from three independent experiments (*n* = 3), each performed in duplicates; p. irr. = post irradiation.

Viability measurements correlated well with diameter measurements of spheroids. As expected, cell viability decreases with increasing radiation doses (Figure 4). Radiation shows dose-dependent growth inhibition in LNCaP and T-47D at all time points, and in PC-3 at 7 and 15 days after irradiation. However, 21 days after irradiation, there is no significant difference between the luminescence signal (viability) of control and irradiated spheroids up to 8 Gy in PC-3. Only after single fraction doses of 20 Gy, viability in PC-3 spheroids is significantly reduced. 

### 3.6. Differences in Metabolic Activity between 2D and 3D (Resazurin Assay)

**Figure 5:**
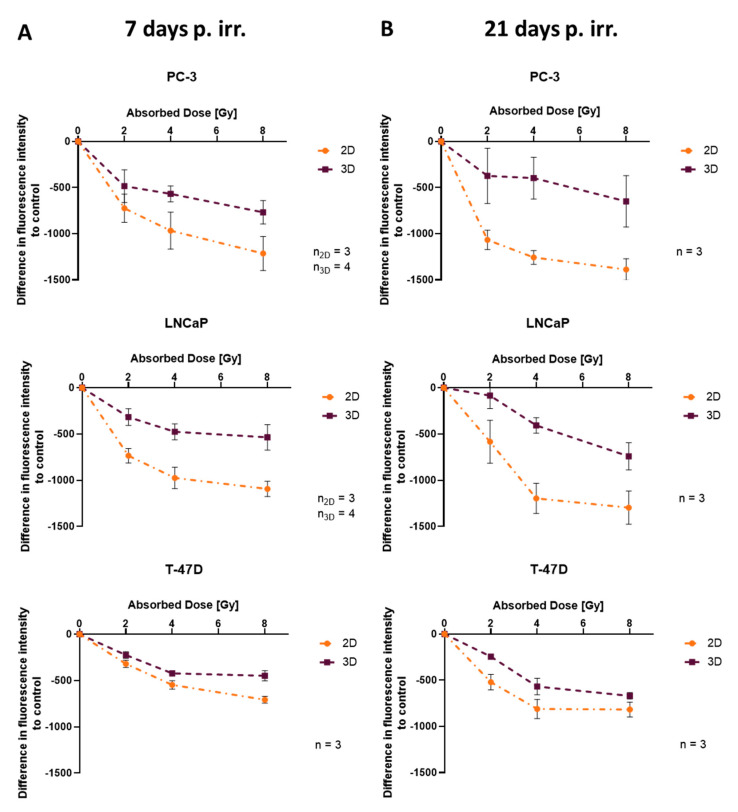
Assessment of difference in fluorescence intensity (metabolic activity) to control between 2D and 3D (**A**) 7 and (**B**) 21 days after irradiation; plotted as mean ± standard deviation from three to four independent experiments (*n* = 3–4), each performed in triplicate; p. irr. = post irradiation.

To compare the radiation response in 2D and 3D using exactly the same method, the resazurin assay was performed. Differences in metabolic activity (measured as fluorescence intensity) to control were higher in 2D than in 3D for all cell lines and all radiation doses. This difference further increased with dose and time (Figure 5A,B). 

### 3.7. DNA Damage Assessment: γH2AX Directly after Irradiation

**Figure 6:**
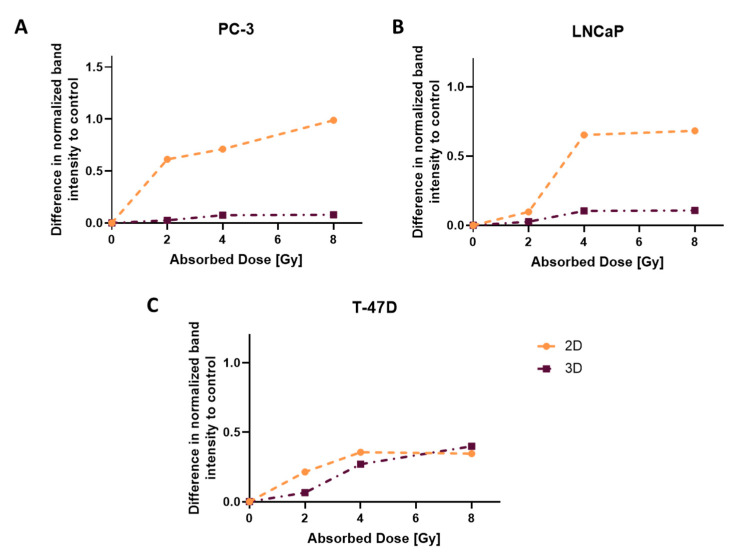
Differences in normalized γH2AX band intensity to control, compared between 2D and 3D directly after irradiation for (**A**) PC-3, (**B**) LNCaP and (**C**) T-47D.

By measuring the amount of double-strand breaks, insight into molecular mechanisms is given. As shown in Figure 6, all cell lines show an increase in γH2AX with increasing radiation doses. Furthermore, more γH2AX was detected in 2D compared to 3D (except for 8 Gy in T-47D), with highest differences in PC-3. Corresponding western blots are depicted in the (Supplementary Figure S5).

## 4. Discussion

In recent years, there has been increasing interest in implementing three-dimensional cell cultures as tumor model systems, being superior to 2D cell cultures when compared to in vivo models. However, there is still a lack of systematic, long term radiobiological studies and, until now, no regard to differences between 2D and 3D cell cultures in the widely used prostate cancer cell lines PC-3 and LNCaP, as well as the breast cancer cell line T-47D. In this study, a radiation response of up to 20 Gy was compared between monolayers and spheroids of these three different human cancer cell lines. 2D and 3D cell culture models were cultivated, treated with X-rays (1, 2, 4, 6, 8 and dose escalation to 20 Gy) and examined on growth behavior, amount of double-strand breaks and viability, using a range of assays. Distinct responses to radiation were shown not only by microscopic growth analysis, but also on various molecular levels.

The Trypan Blue Exclusion assay in monolayers showed no cell death upon irradiation up to 72 h, but, rather, impeded cell replicability in a dose-dependent manner. Single-cell replicability was further investigated by the commonly used clonogenic assay. Resulting data clearly supported a dose-dependent effect: a decrease in cell proliferation with increased radiation dose was observed in all cell lines in 2D. This effect was also shown for LNCaP and T-47D in 3D by means of growth analysis and was also supported by Cell Titer Glo^®^ 3D assay. Similar observations have been made for 3D cultures of HCT116 and CAL27 cells [18]. Regarding the PC-3 cell line, this effect was only observable when comparing day 7 and day 15 after irradiation. 21 days after irradiation, PC-3 spheroids irradiated with up to 8 Gy showed no significant difference in size or viability, compared to the control, suggesting radioresistance up to 8 Gy.

The amount of DNA double-strand breaks correlates with viability data and size measurement of spheroids. Under the same experimental conditions, spheroids showed less double-strand breaks than monolayers. This is in agreement with the findings of Fazeli et al. in the prostate cancer cell line DU145 using Comet Assay [19]. The exception being T-47D spheroids and monolayers, which seem to have similar amounts of double-strand breaks after 8 Gy irradiation.

Comparing dimensionality, the resazurin assay showed big discrepancies between 2D and 3D in all cell lines, which increase with time. The monolayers showed higher difference in metabolic activity to the control, compared to spheroids for all radiation doses, supporting earlier described lower radiosensitivity in spheroids compared to monolayers [13,14,15,16,17,18,19]. Furthermore, the results from the resazurin assay support the data obtained from growth analysis and viability assays: dose-dependent decline was observed in all cell lines 7 days after irradiation, but no significant difference appeared between 2, 4 and 8 Gy in PC-3 spheroids 21 days after irradiation (day 29).

Moreover, clonogenic survival and growth data provided valid indication of cell line specific treatment sensitivity. Our results evinced the highest radiosensitivity for LNCaP (steepest survival curve), followed by T-47D and PC-3 in 2D, and an order of T-47D (significant growth inhibition already with 2 Gy, most-significant difference to control at low doses), LNCaP and PC-3 in 3D. These results raise the knowledge on the differences in radiosensitivity not only between cell lines, but also regarding dimensionality.

Our study supports previous studies, adding data of a bigger repertoire of assays for therapeutic efficacy testing of radiation treatment as well as long term (up to 29 days) radiation response data for the widely used prostate cancer cell lines LNCaP and PC-3 and the breast cancer cell line T-47D. With this, we want to inspire further investigations to gain deeper knowledge on the biological effects of radiation.

However, a limitation of this study is the use of a single cell type in the 3D model. We envisioned utilizing a co-culture model comprising stromal, endothelial and immune cells. Co-culturing aims for better recapitulation of in vivo conditions, such as intercellular interactions between different cell types not represented in the current model. It also remains to be shown how fractionation affects treatment outcome, since this is the current clinical practice in cancer patients treated with external beam therapy, with the general trend of increasing doses per fraction (hypofractionation) enabled by precision radiotherapy techniques. Besides radio-oncological applications, a future step should also be the translation of the current findings into a setup of radionuclide therapy dosimetry.

## 5. Conclusions

This study demonstrates that the radiobiological response to X-rays measured in 2D is not reflected in 3D. The spheroid model shows higher radioresistance in all cell lines, compared to the respective monolayers. This effect is immense when using PC-3 cells, which showed resistance to X-ray radiation up to 8 Gy in spheroids, whereas a dose-dependent decrease in cell proliferation was shown for monolayers. Furthermore, the order of radiosensitivity was determined, observing highest radiosensitivity in LNCaP, followed by T-47D and PC-3 in 2D, but reversed order between LNCaP and T-47D (highest radiosensitivity in T-47D) in 3D. To further foster the understanding of radiobiology in radiation oncology and radionuclide therapy, more in vitro studies need to be performed with special regard to co-culturing methods.

## Figures and Tables

**Table 1:** Coefficients obtained from the linear quadratic curve fitting to measured data.

Cell Line	α (Alpha)	β (Beta)
PC-3	0.165	0.013
LNCaP	0.532	0.236
T-47D	0.199	0.017

## Data Availability

Not applicable.

## Supplementary Materials

The following supporting information can be downloaded at: https://www.mdpi.com/article/10.3390/cells12030360/s1, Figure S1: Growth analysis of spheroids LNCaP and T-47D + Geltrex after single irradiation with 0, 2, 4, 8 or 20 Gy on day 8 (Irr 0); Figure S2: Representative clonogenic assay evaluation; Figure S3: Time lapse growth analysis; Figure S4: Live/dead stainings of spheroids; Figure S5: Western Blots.
