# Supplementary material for: Comparison of Radiation Response between 2D and 3D Cell Culture Models of Different Human Cancer Cell Lines
Source: Cells. 2023 Jan 18;12(3):360. doi: 10.3390/cells12030360 (PMC9913494; doi:10.3390/cells12030360)

Supplementary information

Figure S1. Growth analysis of spheroids LNCaP and T-47D + Geltrex after single irradiation with 0, 2, 4, 8 or 20 Gy on day 8 (Irr 0). (**A**) Time lapse images of LNCaP + Geltrex (**B**) Time lapse images of T-47D + Geltrex (**C**) Diameter of spheroids; average over three diameter measurements per spheroid and time point plotted as mean ± standard deviation from three independent experiments (n = 3); Irr 0, 7, 15 and 21 indicate the time after irradiation in days

(A)

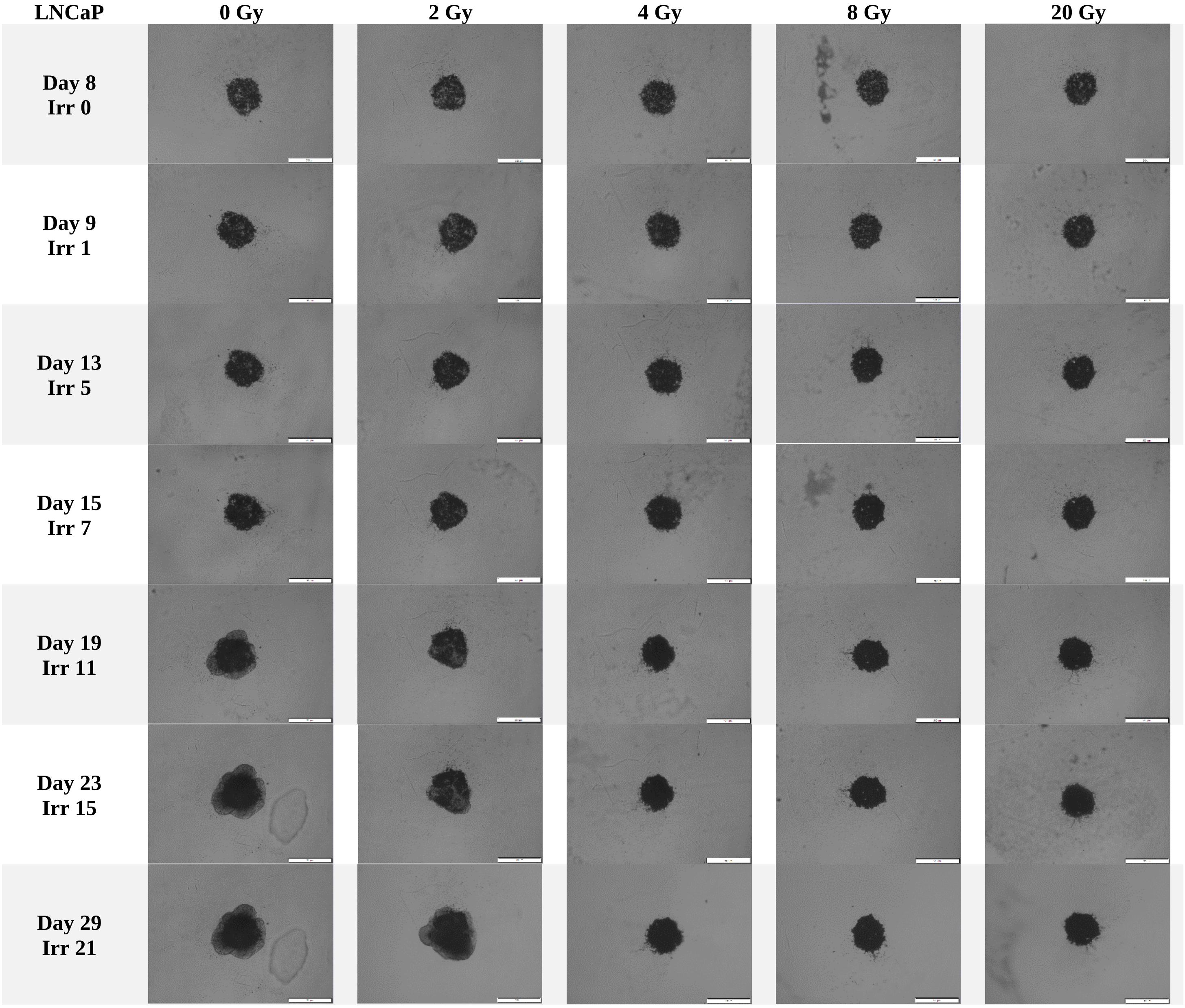

(B)

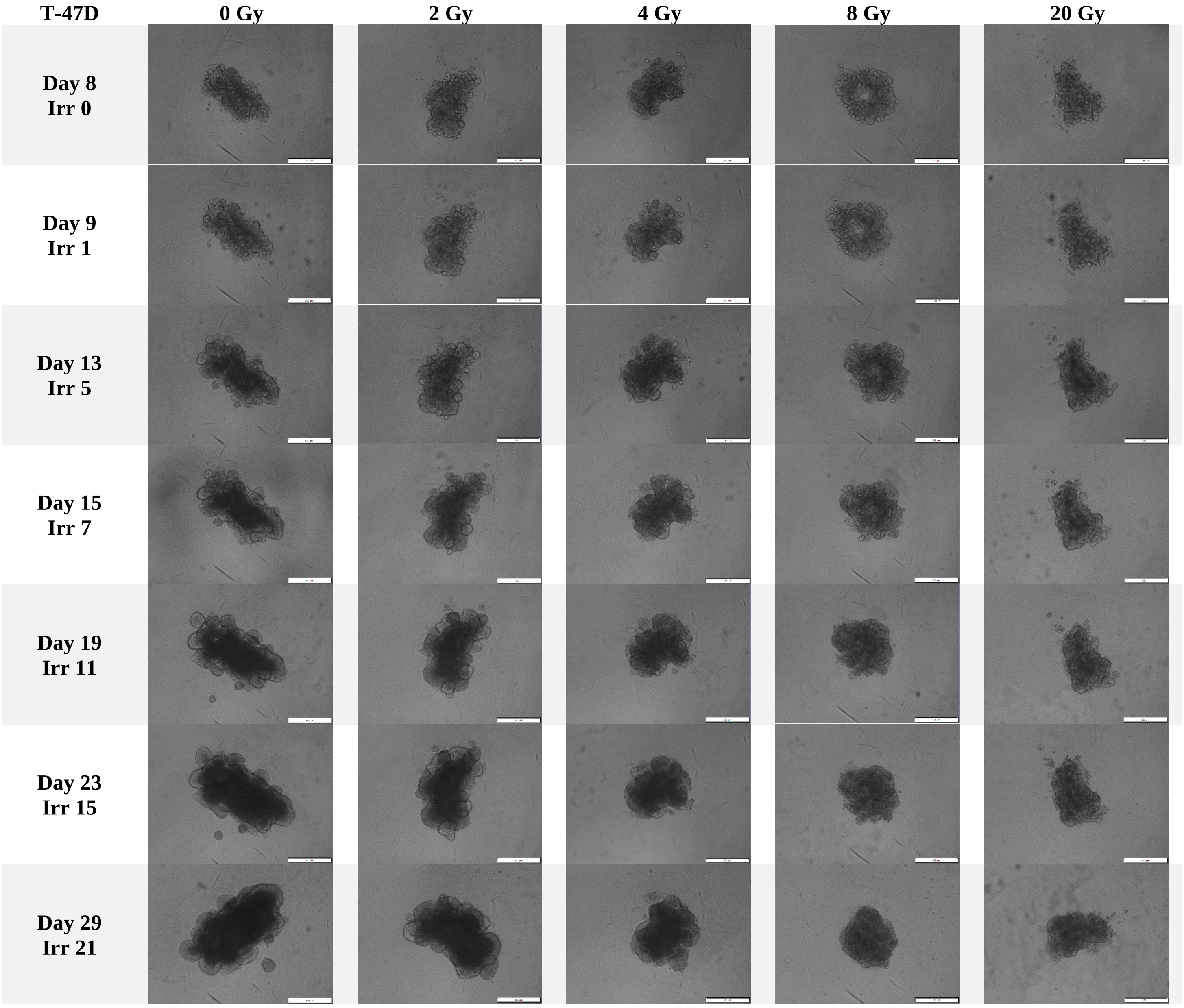
T-47D
0 Gy
2 Gy
4 Gy
8 Gy
20 Gy
Day 8 Irr 0
Day 9 Irr 1
Day 13 Irr 5
Day 15 Irr 7
Day 19 Irr 11
Day 23 Irr 15
Day 29 Irr 21

(C)

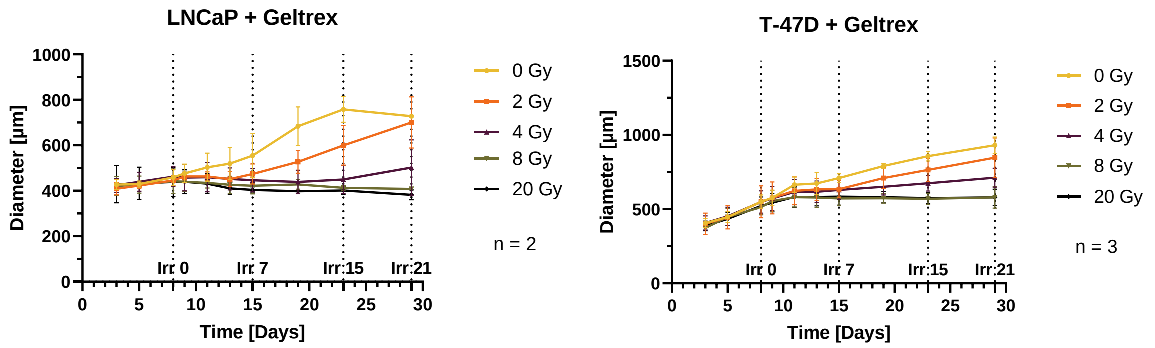
LNCaP + Geltrex
Diameter [µm]
Time [Days]
Irr 0
Irr 7
Irr 15
Irr 21
0 Gy
2 Gy
4 Gy
8 Gy
20 Gy
n = 2
T-47D + Geltrex
Diameter [µm]
Time [Days]
Irr 0
Irr 7
Irr 15
Irr 21
0 Gy
2 Gy
4 Gy
8 Gy
20 Gy
n = 3

Figure S2. Representative clonogenic assay evaluation of (A) PC-3, (B) LNCaP and (C) T-47D wells irradiated with 0, 1, 2, 4, 6 or 8 Gy

(A)

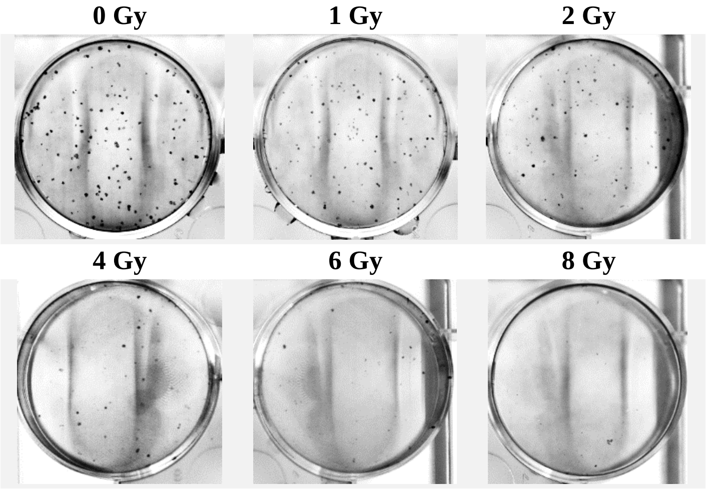

(B)

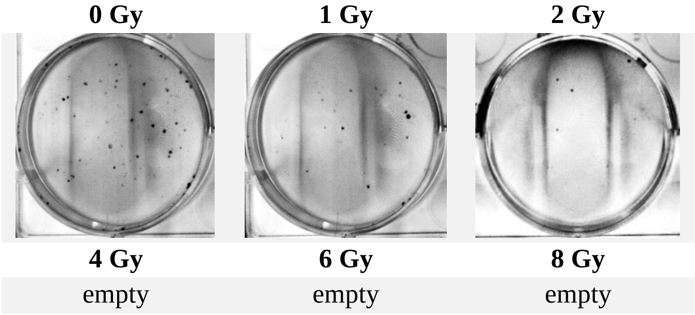

(C)

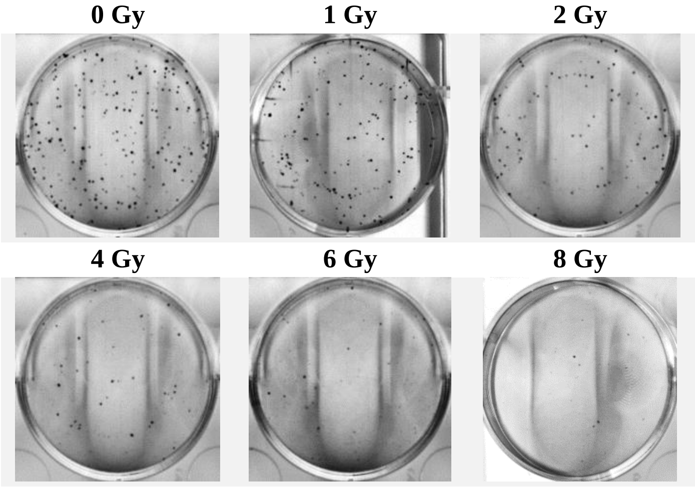

Figure S3. Time lapse growth analysis of (A) PC-3 (with Geltrex), (B) LNCaP and (C) T-47D irradiated with 0, 2, 4, 8 or 20 Gy on day 8 (Irr 0); white scale indicates 500 µm

(A)

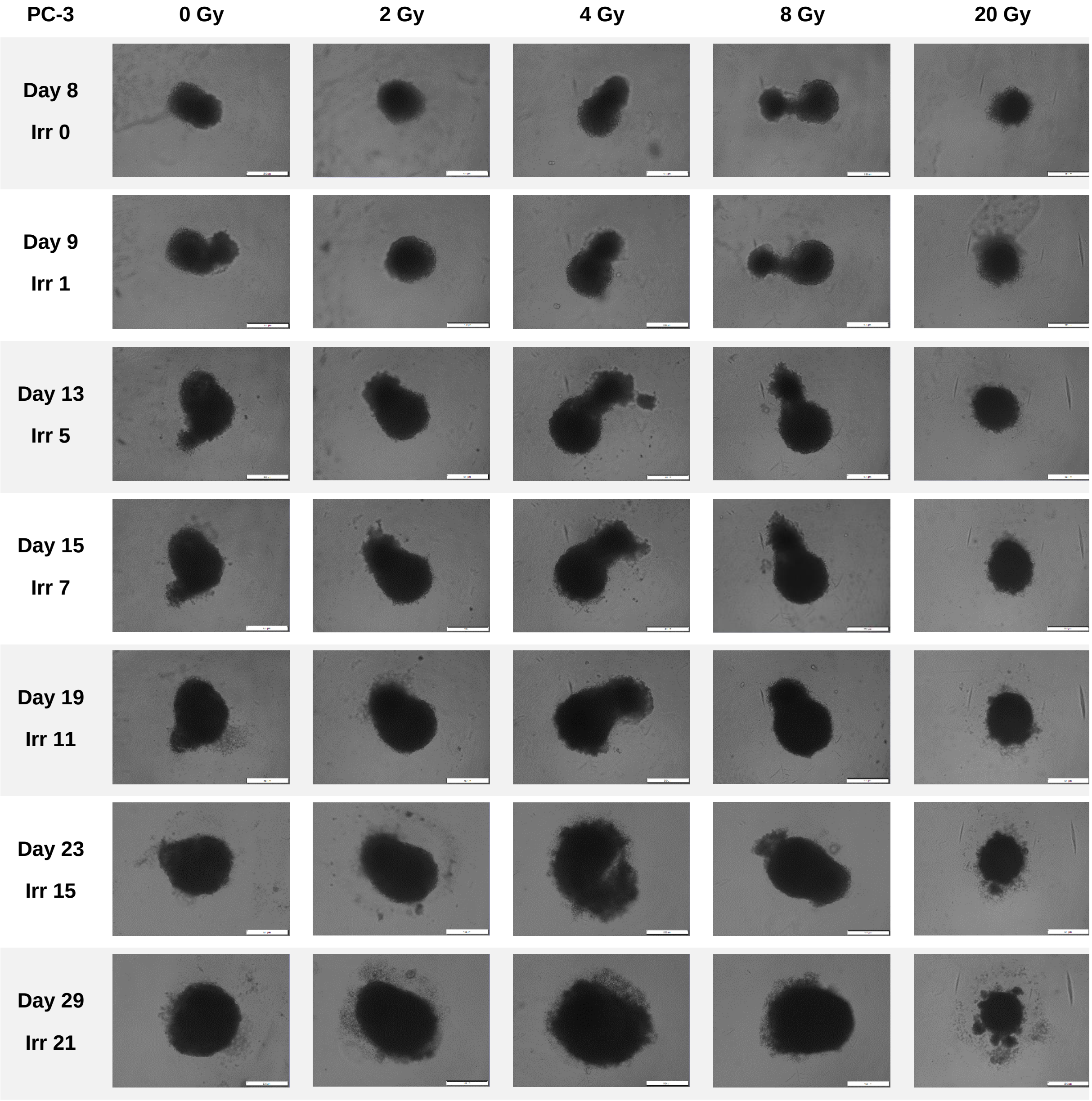

(B)

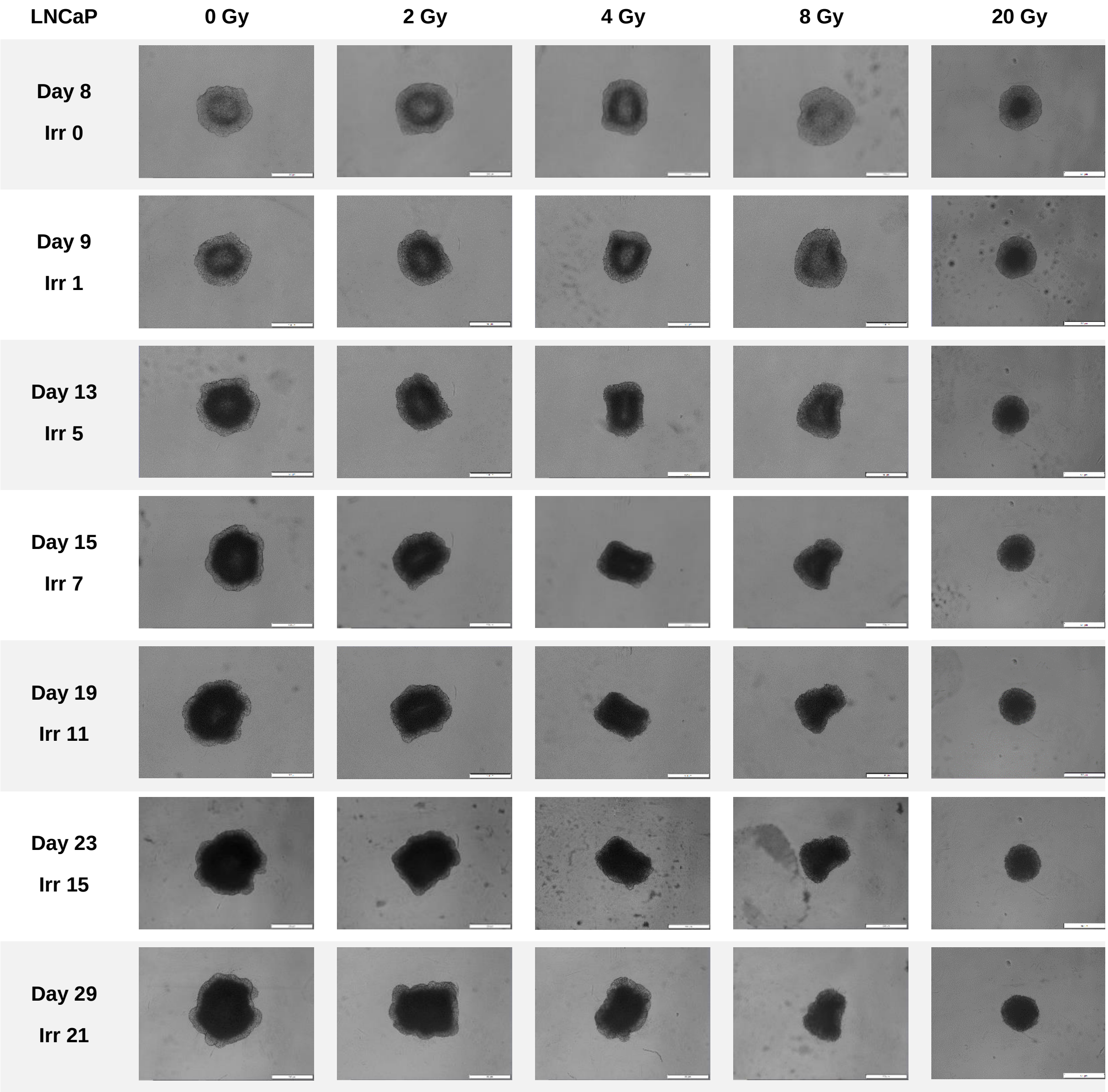
LNCaP
0 Gy
2 Gy
4 Gy
8 Gy
20 Gy
Day 8
Irr 0
Day 9
Irr 1
Day 13
Irr 5
Day 15
Irr 7
Day 19
Irr 11
Day 23
Irr 15
Day 29
Irr 21

(C)

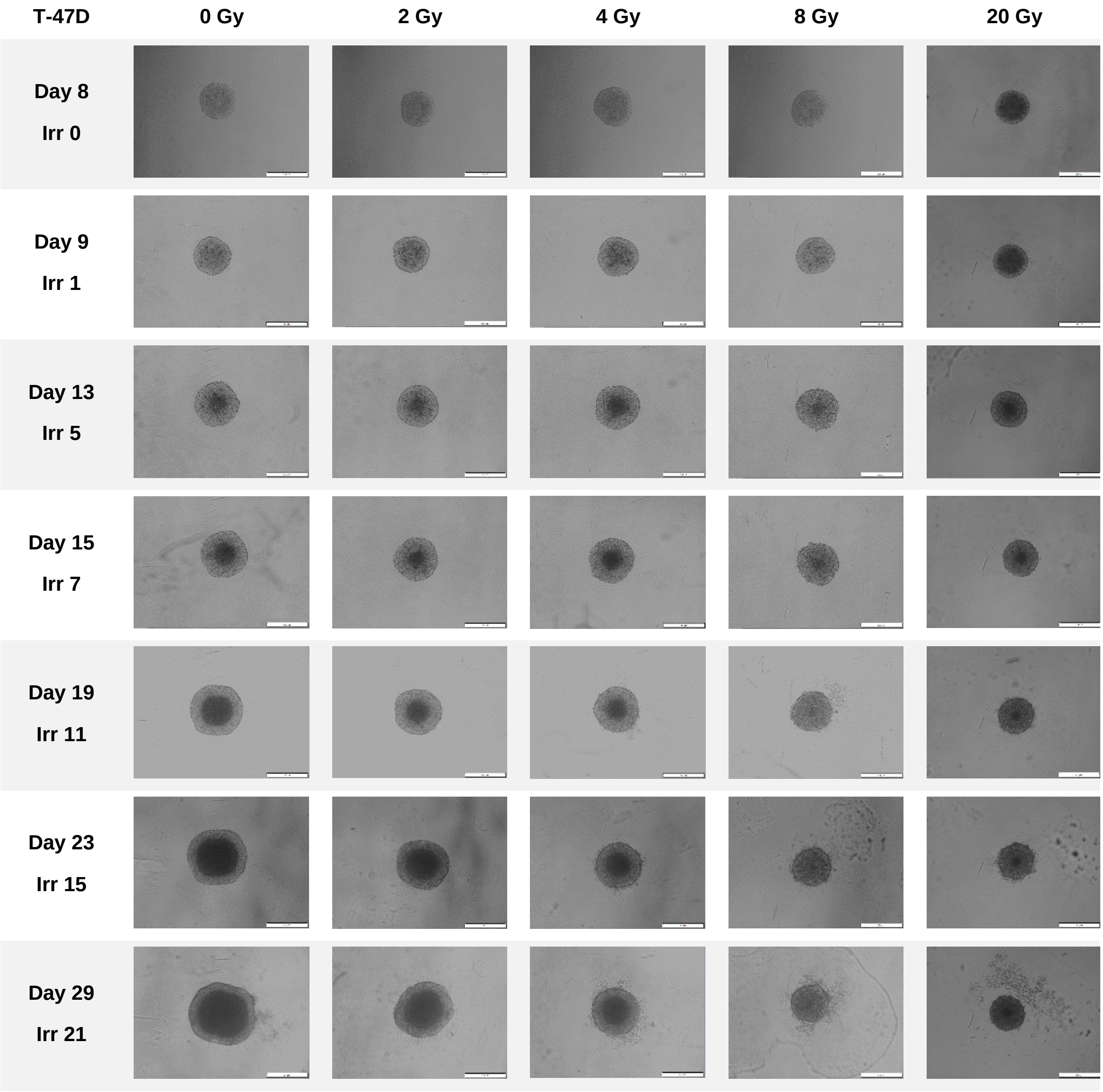
T-47D
0 Gy
2 Gy
4 Gy
8 Gy
20 Gy
Day 8
Irr 0
Day 9
Irr 1
Day 13
Irr 5
Day 15
Irr 7
Day 19
Irr 11
Day 23
Irr 15
Day 29
Irr 21

Figure S4. Live/dead stainings of spheroids. (**A**) Propidium iodide (red) and calcein-AM (green) staining of spheroids on Day 8 (Irr 0), Day 15 (Irr 7) and Day 29 (Irr 21); white scale indicates 500 µm (**B**) Ratio necrotic core to total spheroid size for PC-3, LNCaP and T-47D; plotted as mean ± standard deviation from one or two independent experiments (n=1-2)

(A)

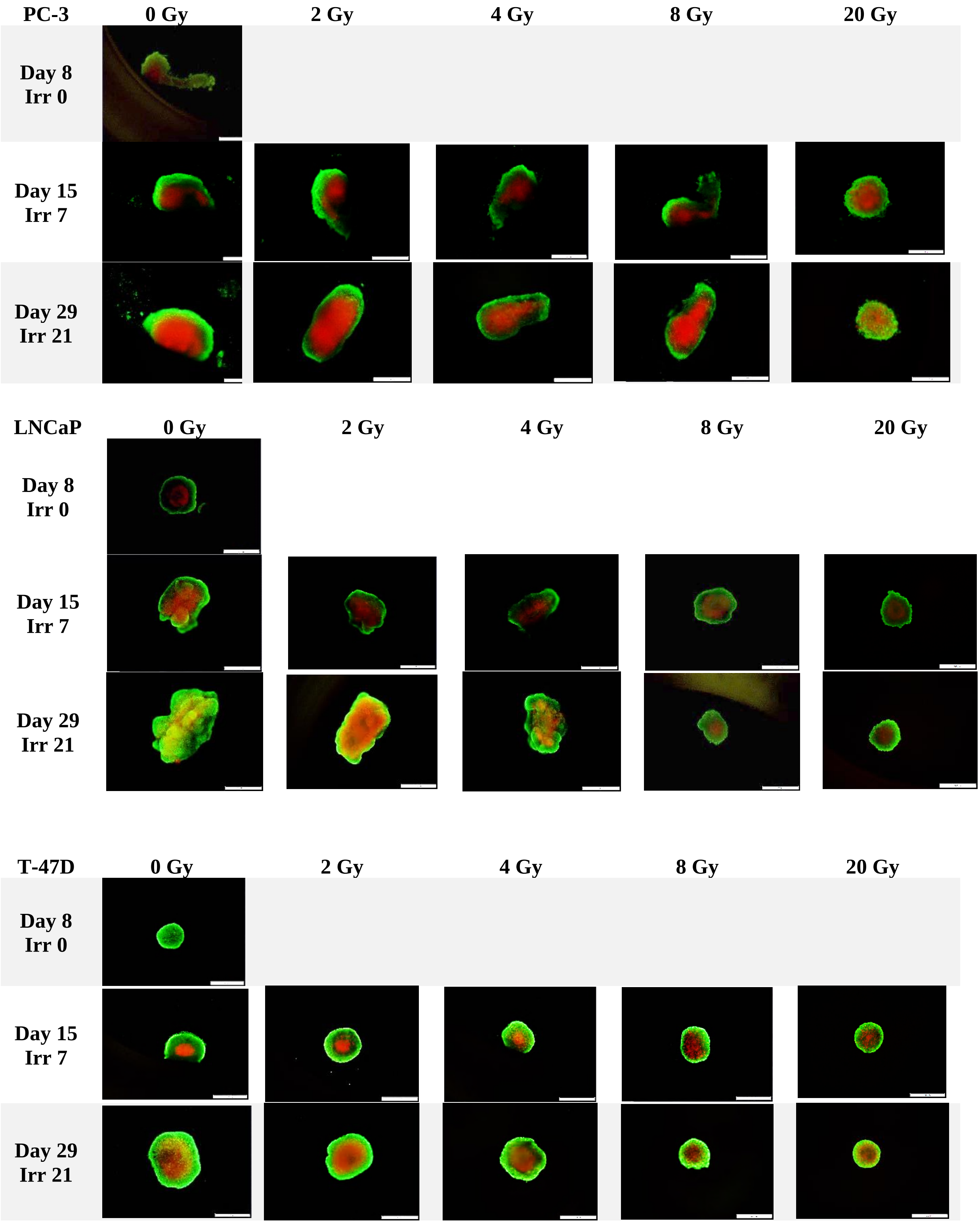

(B)

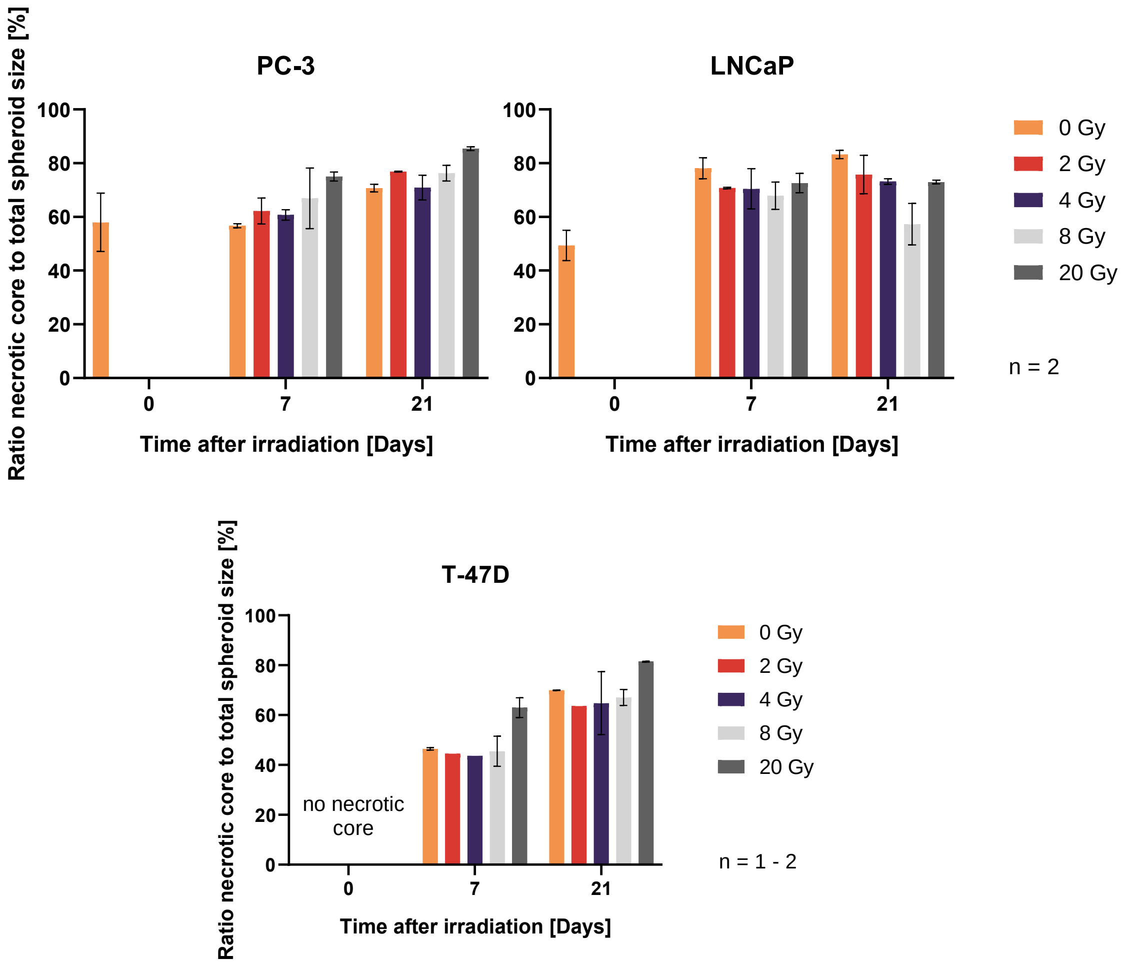
PC-3
LNCaP
T-47D
Ratio necrotic core to total spheroid size [%]
100
80
60
40
20
0
0
7
21
Time after irradiation [Days]
0 Gy
2 Gy
4 Gy
8 Gy
20 Gy
n = 2
no necrotic core
n = 1 - 2

Figure S5. Western Blots for further analysis with ImageJ

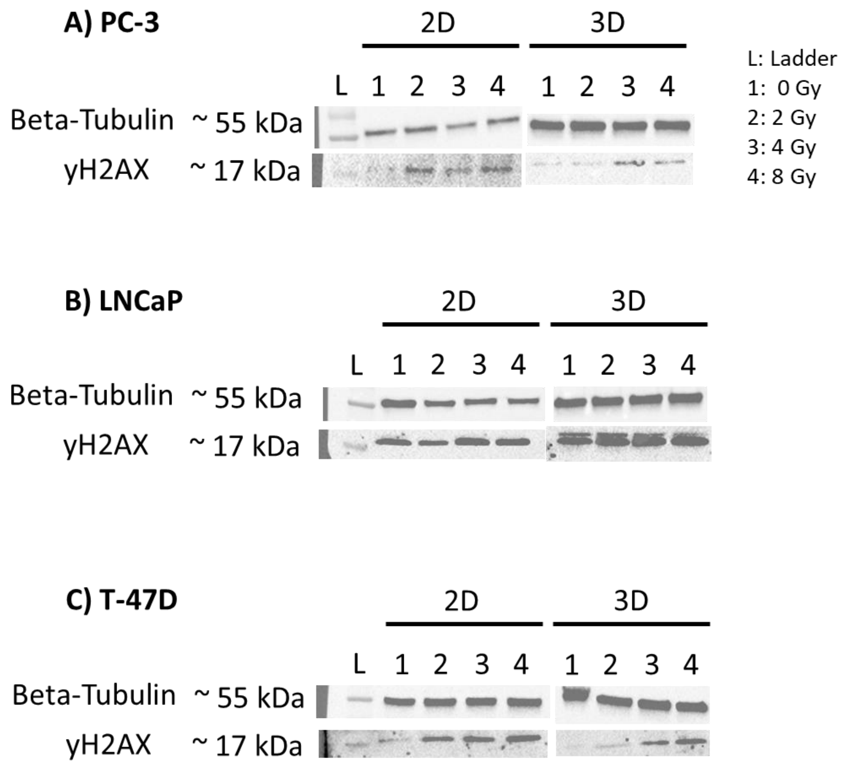

Supplement: Supplementary file 1 [file cells-12-00360-s001.zip › cells-2079002-supplementary.pdf]
